# Brown-to-white transition in subcutaneous fat: linking aging and disease

**DOI:** 10.18632/aging.100509

**Published:** 2012-12-07

**Authors:** Nicole H Rogers, Roy G Smith

**Affiliations:** Department of Metabolism and Aging, Scripps Research Institute Florida, Jupiter, FL 33458

Insulin resistance increases with age, but molecular mechanisms remain unclear. Aging causes loss of subcutaneous white adipose tissue (sWAT) associated with a redistribution of fat from peripheral to central stores [1]. Interestingly, in older individuals the ratio of peripheral/central fat is a stronger determinant of insulin resistance than central adiposity alone [2]. This suggests sWAT is likely to be important, but age-dependent alterations specific to this depot remain poorly characterized; therefore, we recently investigated how murine sWAT changes as a function of age [3].

**Figure 1:**
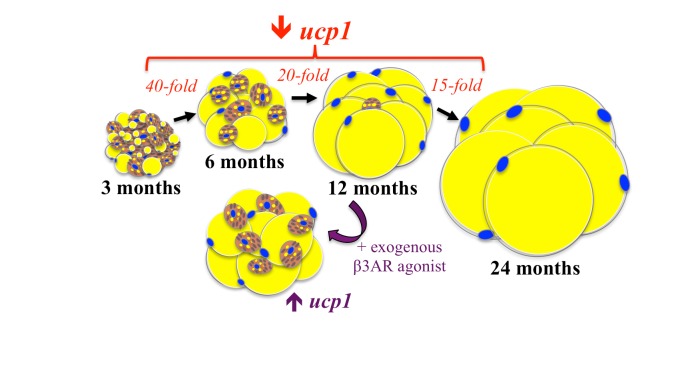
Brown-to-white transitioning and loss of *ucp1* expression in murine sWAT during aging At 3 months of age, numerous brown adipocytes reside in sWAT and *ucp1* expression is high. However, by 6 months of age, there are fewer brown adipocytes and expression of *ucp1* is reduced nearly 40-fold. By 12 months, the depot appears uniformly white and *ucp1* levels have decreased an additional ~20-fold (vs 6 mo). Expression of *ucp1* then continues to fall another ~15-fold between 12 and 24 months. When 12 month old animals are treated with a β-3 adrenergic receptor (β3AR) agonist for seven days, brown adipocytes reappear and *ucp1* expression is increased.

A unique feature of sWAT is the presence of highly oxidative brown adipocytes. However, our recent studies demonstrate an age-dependent disappearance of these brown adipocytes associated with the development of insulin resistance [3]. In murine sWAT, we observe a precipitous decline in expression of *ucp1*, the hallmark of a brown adipocyte, with advancing age (Figure 1). Remarkably, *ucp1* levels decrease more than 800-fold between 3 and 12 months of age (Figure 1). Loss of sWAT ‘browning’ with age is further supported by reduced expression of other brown-indicator genes, including *cidea, cox7a1*, and *ppara*, as well as corresponding histological changes.

What initiates this brown-to-white transition remains unclear, but adiposity is unlikely because animals that do not gain weight while aging display similar loss of *ucp1*. Older animals have reduced adrenergic receptor expression and increased expression of enzymes that degrade catecholamines, suggesting local adrenergic tone is a factor. Indeed, our unpublished findings reveal age-dependent alterations in a number of additional modulators of adrenergic signaling. Further, we show that administering an exogenous β-3 adrenergic receptor (β3AR) agonist effectively reverses the phenotype (Figure 1). Ongoing studies are directed towards a more detailed understanding how adrenergic signaling and other mechanisms promote these age-dependent changes in the phenotype of sWAT. Additional interesting questions generated by the findings include:

1. *Do brown-like characteristics in human sWAT similarly decline with age?* Cold-induced activity of brown adipose tissue found in humans is reduced with age [4]. Interestingly, human brown adipose tissue displays more similarities to murine ‘beige’ fat, or the brown adipocytes residing in sWAT, than classical brown fat [5]. *Ucp1* is expressed in human sWAT [6], hence, evaluating age-dependent variation and its potential link with metabolic diseases will be important.
2. *Is sWAT expression of ucp1 associated with diabetes status as humans age*? Our studies reveal an inverse association between sWAT expression of *ucp1* and hyperglycemia in older mice. It has been reported that obese humans with diabetes have less *ucp1* in sWAT than obese humans without diabetes, suggesting brown-like characteristics of sWAT are protective [6]. Perhaps older individuals that become insulin resistant have fewer brown adipose tissue markers in sWAT than those that remain insulin sensitive.
3. *Is it possible to preserve insulin sensitivity during aging by maintaining brown-like characteristics in sWAT?* Our findings highlight reversibility in the phenotype, but whether increasing *ucp1* specifically in sWAT would preserve insulin sensitivity remains unclear. Caloric restriction is an appreciated means of increasing insulin sensitivity with age, but surprisingly we do not observe dramatic differences in expression of *ucp1* in sWAT from calorie restricted mice. However, other genes preferentially expressed in brown adipocytes, such as *cidea* and *ppara*, are better maintained in sWAT with caloric restriction. This dissociation in the expression of ‘brown-marker’ genes suggests adipocytes are actually various shades of brown, and further, factors such as energy balance are able to compensate for a programmed loss of UCP1+ adipocytes by altering the oxidative phenotype of other adipocytes.

While more work is necessary to fully understand the implications for human aging and disease, taken together, our findings reveal novel molecular events related to the development of insulin resistance during aging and highlight sWAT as a relevant therapeutic target.
